# Identification of the main genetic clusters of avian reoviruses from a global strain collection

**DOI:** 10.3389/fvets.2022.1094761

**Published:** 2023-01-12

**Authors:** Edit Kovács, Renáta Varga-Kugler, Tamás Mató, Zalán Homonnay, Tímea Tatár-Kis, Szilvia Farkas, István Kiss, Krisztián Bányai, Vilmos Palya

**Affiliations:** ^1^Ceva-Phylaxia Ltd., Budapest, Hungary; ^2^Veterinary Medical Research Institute, Budapest, Hungary; ^3^Department of Obstetrics and Food Animal Medicine Clinic, University of Veterinary Medicine, Budapest, Hungary; ^4^Department of Pharmacology and Toxicology, University of Veterinary Medicine, Budapest, Hungary

**Keywords:** avian reovirus, global, collection, diversity, co-infection

## Abstract

**Introduction:**

Avian reoviruses (ARV), an important pathogen of poultry, have received increasing interest lately due to their widespread occurrence, recognized genetic diversity, and association to defined disease conditions or being present as co-infecting agents. The efficient control measures require the characterization of the available virus strains.

**Methods:**

The present study describes an ARV collection comprising over 200 isolates from diagnostic samples collected over a decade from 34 countries worldwide. One hundred and thirty-six ARV isolates were characterized based on σC sequences.

**Results and discussion:**

The samples represented not only arthritis/tenosynovitis and runting-stunting syndrome, but also respiratory symptoms, egg production problems, and undefined disease conditions accompanied with increased mortality, and were obtained from broiler, layer or breeder flocks. In 31 percent of the cases other viral or bacterial agents were demonstrated besides ARV. The most frequent co-infectious agent was infectious bronchitis virus followed by infectious bursal disease virus and adenoviruses. All isolates could be classified in one of the major genetic clusters, although we observed marked discrepancies in the genotyping systems currently in use, a finding that made genotype assignment challenging. Reovirus related clinical symptoms could not be unequivocally connected to any particular virus strains belonging to a specific genetic group, suggesting the lack of strict association between disease forms of ARV infection and the investigated genetic features of ARV strains. Also, large genetic differences were seen between field and vaccine strains. The presented findings reinforce the need to establish a uniform, widely accepted molecular classification scheme for ARV and further, highlight the need for ARV strain identification to support more efficient control measures.

## 1. Introduction

Avian reovirus (ARV) infect a wide range of avian species including wild birds and domestic poultry. Although the virus is ubiquitous and commonly isolated from healthy birds, various forms of clinical disease caused by pathogenic ARV strains are described. The most common clinical manifestations of ARV infections in chickens are viral arthritis/tenosynovitis and runting-stunting syndrome, but ARV infection has been associated with uneven growth, poor feed conversion, and increased mortality or morbidity due to secondary infections, which indicates its possible association with immunosuppressive conditions, including ARV among other infectious or non-infectious causes. In addition, ARVs have been associated with various disease conditions such as, hepatitis, myocarditis, diseases of the central nervous system and the immune system (1–5). The consequences of avian reovirus infection are influenced by many factors, such as the age of the host, host immunity, route of infection, pathogenicity of the ARV strain, presence of other infectious agents. The role of ARVs as primary pathogens has been most profoundly investigated and proven in viral arthritis/tenosynovitis in the 1950's. The clinical signs of the disease can appear as early as 1–2 weeks of age. Inflammation of the tibiotarsal-tarsometatarsal joints and rupture of the gastrocnemius tendon cause different severity of lameness either in young broilers or breeder chickens (1). Runting-stunting syndrome was first reported in broilers during the 1970's and was described in different names such as malabsorption syndrome (MAS), brittle bone disease and helicopter wing syndrome. The disease is most frequently diagnosed in 2–3 weeks old chickens, and characterized by slow development, uneven growth rate, bone formation disorder and abnormal feathering. Among the numerous viruses that have been associated with the disease, ARV is one of the most frequently implicated pathogens of this complex etiology (6).

ARVs are medium-sized, non-enveloped icosahedral virion enclosing 10 double-stranded RNA genome segments. The segments are classified into three size classes, such as L (large, containing L1 to L3 segments), M (medium, M1–M3) and S (small, S1–S4), and they encode eight structural and three or four nonstructural proteins. The structural protein σC is responsible for cell attachment and virus neutralization, consequently, σC is key target of routine epidemiological investigations and the primary virus antigen for vaccine development. The coding sequence of σC has been used to create classification schemes of ARV into genogroups. Kant et al. (7) have defined five clusters based on the partial sequence of the S1 genome segment that encodes σC protein. When additional strain diversity has become evident, new typing schemes were proposed, but these new typing schemes were often controversial because they used different sequence sets and analysis tools, used a given cluster name interchangeably that prevented the introduction of a uniform σC based classification scheme for ARVs (8, 9).

Since the virus can be transmitted both vertically and horizontally, the control of ARV-associated diseases is based on vaccination of both breeders and broilers with live attenuated and/or inactivated whole virus vaccines. The classical vaccine strains S1133, 1733, and 2408 were isolated in the USA in the 1970's (30). The genetic and antigenic diversity of field strains makes the control of ARV by vaccination very challenging. From the 2010's onward, increasing number of tenosynovitis cases have been reported in vaccinated poultry flocks worldwide (2, 5, 8, 9). Novel, emerging variants of ARV have been detected in many of these outbreaks, which were able to escape the immunity induced by the commercially available vaccines. ARV strains causing vaccine breakthrough were found to differ antigenically from the vaccine strains used to manufacture commercially licensed vaccines (5, 10–13). The novel variants have attracted the attention of all participants of the poultry industry, including poultry breeders and poultry vaccine producers. Besides the commercially licensed vaccines, autogenous vaccines may become promising tools to control the novel ARV strains caused losses.

The objectives of the present study were to evaluate the global diversity of ARVs using laboratory records of Ceva-Phylaxia and sequence information of σC coding gene collected during a period of broadly one decade.

## 2. Materials and methods

### 2.1. Samples

The samples submitted to the Scientific Support and Investigation Unit of Ceva-Phylaxia Co. for diagnostic investigation were collected from commercial broiler and layers and breeders originated from a wide geographic distribution. Different types of samples such as bursa, caecal tonsil, cloacal or oronasal swab, eye lid, spleen, liver, pancreas, proventriculus, gizzard, intestine, joint, kidney or trachea were sent from the field according to the clinical symptoms observed.

### 2.2. Virus isolation

The organs sent were minced with sterile scalpel in a BSL-2 biosafety cabinet, then homogenized in PBS containing a mixture of antibiotics with Bullet Blender ® 50-DX Homogenizer at the highest speed for 10 min, then centrifuged at 4,100 rpm for 15 min. The supernatant was filtered through 0.22 μm syringe filter. 0.2 ml of filtered supernatant was inoculated in the allantoic cavity of 9 day-old embryonated SPF eggs. The eggs were candled daily for seven days. 0.1 ml from the allantoic fluid of dead or affected embryos was inoculated on LMH cell culture (ATCC Number CRL-2117) and incubated at 37 °C with 4–5 % CO_2_ in a humidified incubator for 7 days or until the cytopathic effect (CPE) reached 90 percent based on the daily observation. Supernatant of CPE positive LMH cell culture was used as a material for subsequent RT-PCR test. Joint samples were inoculated directly on LMH cell culture after the homogenization and clarification steps. In case of negative result after two blind passages, the joint sample was inoculated into the yolk sac of 9 day-old embryonated SPF eggs and handled as written above.

### 2.3. RT-PCR and σC gene sequences

The presence of ARV in the different samples was checked and quantified by RT-qPCR. RNA was extracted with MagMAX™-96 Total RNA Isolation Kit using MagMax Express 96 MagneticParticle Processor (Thermo Fisher Scientific). The M1 gene-specific, broad range RT-qPCR was carried out according to Tang et al. (Infection, Genetics and Evolution; 2016, 39:120–126).

Sanger sequencing and semiconductor sequencing techniques were utilized to obtain the σC protein coding genomic region.

Viral RNA was extracted using the Direct-zol RNA Miniprep kit (Zymo Research). Oligonucleotide primers (P1 (Fw) 5'-AGTATTTGTGAGTACGATTG-3' and P4 (Rev) 5'-GGCGCCACACCTTAGGT-3') that hybridize to a 1,088 portion of ^(2)^ the sigma C gene of S1 segment were adapted from Kant et al. (7). Amplification was carried out by using the one-step RT-PCR kit of QIAGEN. PCR products were run in low melting point agarose gel stained with ethidium bromide, and then excised and column purified (QIAquick Gel Extraction Kit, QIAGEN). PCR primers were used in the sequencing reaction, which was performed according to the recommendations of the manufacturer (BigDye cycle sequencing kit v3.1; Applied Biosystems). The dye-labeled products were run on an automated sequence analyzer (ABI 3100). After preliminary sequence data were retrieved, additional internal primers were designed to complete the sequences (not shown).

Concerning the semiconductor sequencing, random primed reverse transcription was followed by amplification of complementary DNA (cDNA). A cDNA library was prepared using the NEBNext Fast DNA Fragmentation & Library Prep Set for Ion Torrent (New England Biolabs, Beverly, MA, USA) using the Ion Torrent Xpress Barcode Adapters (Thermo Fisher Scientific, Waltham, MA, USA). The emulsion PCR and subsequent templated bead enrichment was performed with a OneTouch v2 instrument and Ion OneTouch ES (Thermo Fisher Scientific), respectively. Sequencing was carried out on a 316 chip using the Ion Torrent Personal Genome Machine (Thermo Fisher Scientific). Additional details of this viral metagenomics approach are shared elsewhere (14, 15).

### 2.4. Sequence analysis

Raw Sanger sequencing reads were assembled by using BioEdit (16), GeneDoc (17) and MultAlin (18), whereas short-read sequences from Ion Torrent were assembled by using reference sequences within the software QIAGEN CLC Genomics Workbench.

Phylogenetic analysis was performed with the MEGA6 (19) program package based on multiple sequence alignments (768 nt and 256 aa long) generated by the TranslatorX (20) online platform; the best-fit substitution models were selected for each gene-specific dataset based on the Bayesian information criterion. Maximum-likelihood trees were generated, and tree topologies were validated by bootstrap analysis (1,000 replicates). The length of sequences in the final multiple alignments were 768 nucleotides and 256 amino acids. Reference sequences including some of those reported in Kant et al. (7) were used to serve as backbone for genotyping.

## 3. Results

### 3.1. Diagnostic findings

Two hundred and five ARV strains were included in the study. All the isolates derived from diagnostic samples except three vaccine seed strains. Clinical samples were received from 34 countries of four continents (Table 1). The first ARV isolate was obtained in 2002, the second one in 2008. The number of ARV detections increased from 2009 but showed significant fluctuation in the last 10 years. Although various types of organs were submitted for diagnostic investigations, 37 percent of ARV isolates were obtained from caecal tonsil. Twenty-two percent of ARV strains were isolated from the joint. Ten percent of isolates were detected from bursa and 6 percent from the liver samples. The clinical symptoms observed in the flocks from which the submitted samples yielded positive ARV detection varied; about half of the cases were indicative of ARV infection, but in the other part of cases no ARV-related clinical signs were reported.

**Table 1:** Distribution of the clusters based on the diseases and geographical area.

	**TS**	**RS**	**TS/RS**	**O**	**ND**	**V**	**Sum**	**Africa**	**America**	**Asia**	**Europe**	**Middle East**
Cluster 1	9	2	0	12	3	1	27	0	4	9	10	4
Cluster 2	4	7	1	18	6	1	37	2	5	13	12	5
Cluster 3	1	1	0	9	2	0	13	2	1	4	3	3
Cluster 4	12	10	1	29	12	0	64	2	8	13	32	9
Cluster 5	4	3	0	1	1	0	9	1	2	2	2	2
n.a.	31	9	0	11	3	1	55	0	6	31	17	1

TS, tenosynovitis/arthritis; RS, runting-stunting syndrome; O, other; ND, not defined; V, vaccine.

Most of the samples originated from Asia and Middle East (72 and 24 samples, respectively), 76 samples were submitted from Europe, 26 samples from America, while the lowest number of samples were received from Africa (*n* = 7). There was no submission from Australia to our laboratory.

**Figure 1:**
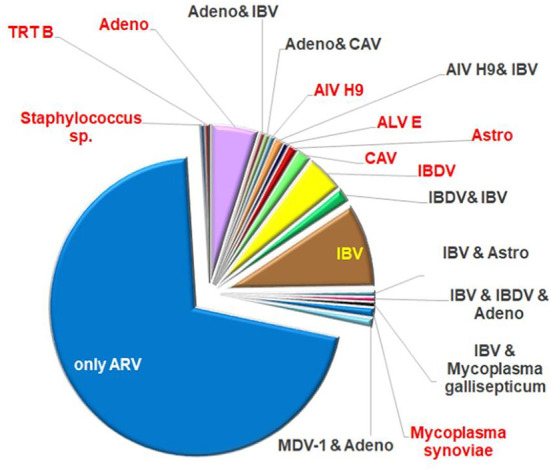
Ratio of co-infecting agents in the field samples.

ARV was identified as the only infective agent in 69% of the cases of this database. Two or three different ARV strains were present in the same sample in 13 cases. Thirty-one percent of the investigated submissions proved to be a mixed infection with other agents. Only one other pathogen was demonstrated in 47 samples, while two or three pathogens were detected beside the ARV strain in 12 samples. Most frequently, IBV was detected as a co-infecting virus, furthermore in a moderate number IBDV and adenoviruses were found in the diagnostic samples. Other viruses, such as LPAIV (low pathogenic avian influenza virus) H9, TRTV (turkey rhinotracheitis virus), MDV (Marek's disease virus), ALV (avian leucosis virus) E, chicken astrovirus, and CAV (chicken infectious anemia virus) were identified in few cases and bacterial infections: Mycoplasma gallisepticum, Mycoplasma synoviae and Staphylococcus species were present in four clinical samples (Figure 1). It should be noted that the bacteriological investigations were performed only on request.

**Figure 2:**
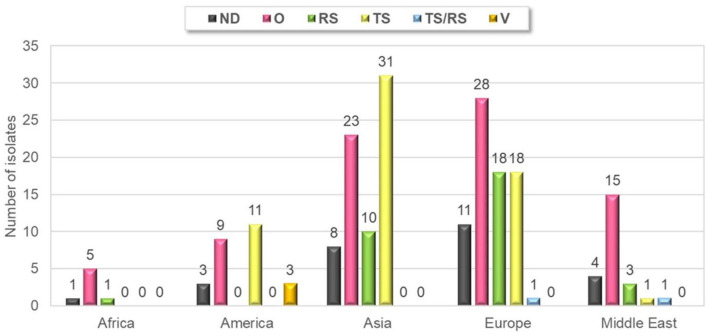
Geographical distribution of different clinical manifestations of submissions. TS, tenosynovitis/arthritis; RS, runting-stunting syndrome; O, other; ND, not defined; V, vaccine.

Forty-six percent of strains were isolated from samples originating from chickens showing arthritis/tenosynovitis (61 cases) or runting–stunting syndrome (32 cases) and there were two cases, where both ARV specific clinical signs were present at the same time. Thirty-nine percent of the isolates were detected from clinical cases with non-ARV specific symptoms such as increased mortality and respiratory symptoms with or without other clinical or gross-pathological signs and gout, kidney lesions, egg production or eggshell problems and bacterial infections. Information about the clinical symptoms was not disclosed in 13 percent of the isolates. Arthritis-tenosynovitis and runting-stunting syndrome were observed in each of continents included in this study, but no sample was submitted from the runting-stunting syndrome from America (Figure 2).

Clinical samples were collected from chickens aged 6 days to 39 weeks. The age of the sampled bird was not disclosed in 23 clinical cases. From chickens showing symptoms of arthritis-tenosynovitis ARV isolates were obtained from chickens aged 7 days to 28 weeks, but most of the positive samples originated from 5 to 6 weeks old birds. ARV strains causing runting-stunting syndrome were isolated from chickens aged 9 days to 56 days.

### 3.2. Strain characterization

A total of 136 isolates (representing 71% of all isolates in the strain collection) were subjected to further sequence analysis. The partial σC coding gene was amplified and sequenced by the Sanger method for all strains; of these, 123 gave unambiguous sequences. In another 13 cases, low quality sequence chromatograms prevented any downstream analyses. Instead of molecular cloning of the σC PCR product, we used viral metagenomics approach. In all 13 cases definite sequence variants could be distinguished, which were assigned as VAR1 and VAR2 and, sometimes, VAR3 for the respective dual or triple isolates (see **Figure 6** for details). Each (including VAR1, VAR2, and VAR3) final consensus nucleotide sequences were aligned and translated into an amino acid-based alignment. The phylogenetic tree was generated from these deduced amino acid sequences.

In general, the analysis included some key reference strains from Kant and co-workers' 5-cluster system as well as some vaccine strain sequences and showed that all strains can be assigned into a cluster within this classification system. Clusters 1 to 5 included the sequences of 27, 37, 13, 64, and 9 study strains, respectively, including 27 sequences from 13 mixed infections. Noticeably, in mixed infections, the identified two or three strains typically belonged to different clusters. This pattern was seen in 11 of 13 mixed infections and only 2 isolate had different sequences from the same cluster.

Using this set of sequences, the within cluster amino acid sequence identities were found to fall in the range of 52 to 100% with lowest minimum values in clusters 3 and 4. Inter-cluster similarities were lower than 62% and were, in some cases, as low as 39%.

Cluster 4 was present in the highest proportion (64 isolates, 31%). Twenty-seven isolates (13%) were classified into cluster 1, which also includes the commercially available vaccine strains S1133, 1,733, and 2,408. Thirty-seven isolates (18%) belonged to cluster 2. Cluster 3 was found in 6 percent of the isolates and cluster 5 was present only in 4 percent of the isolates. Twenty-seven percent of the isolates were not genetically identified (i.e., could not be sequenced).

**Figure 3:**
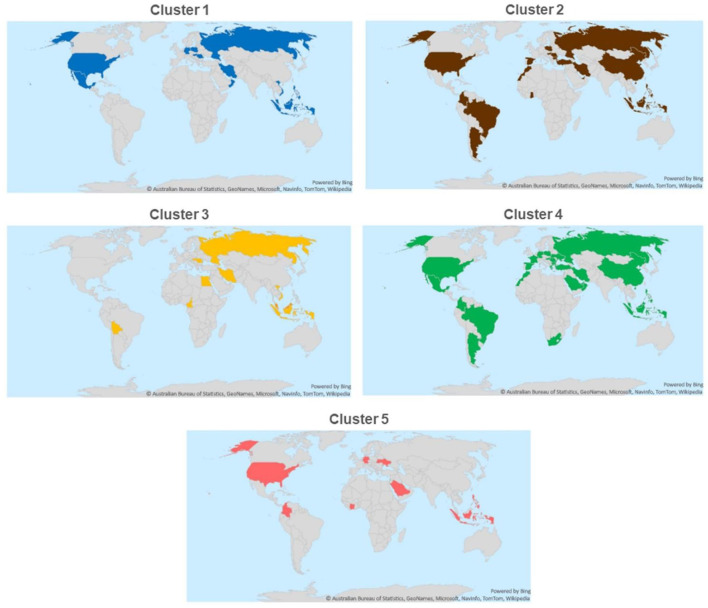
Geographical distribution of different ARV clusters.

**Figure 4:**
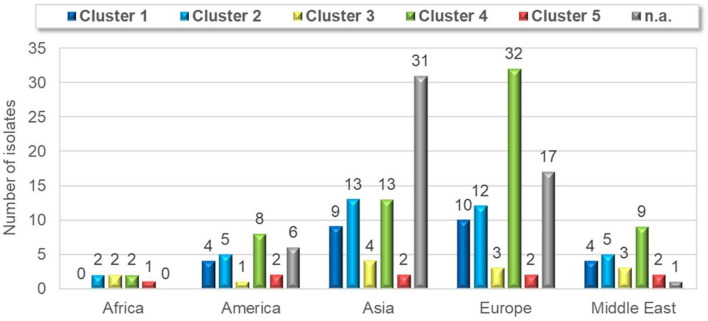
Geographical distribution of different ARV clusters with the numbers of cases.

**Figure 5:**
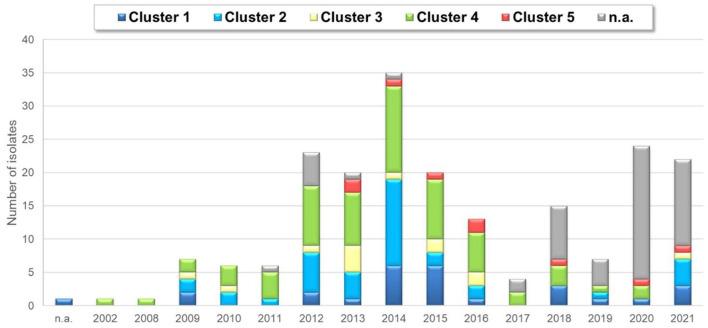
Yearly distribution of ARV detection based on clusters.

Strains belonging to each of the five clusters were detected on each continent except Africa where cluster 1 was not identified from the 7 isolates analyzed. Cluster 4 isolates were the most frequently detected cluster in each continent, while cluster 3 and cluster 5 isolates were present in the lowest ratio. Cluster 5 strains appeared only in the sample collected after 2013 (Figures 3–5). Percent identities among strains were highest when strains were identified in the same geographic region (e.g., some German and Mexican strains within cluster 1, Malaysian, Spanish and Russian strains within cluster 4, and German and US strains within cluster 5; up to 100% identity in respective sequence comparisons).

Within cluster 2, Asian strains were closely related to each other within two independent lineages while another lineage contained European strains from the Balkan peninsula and neighboring countries. A single American and three independent African cluster 2 isolates with moderate sequence identities were also found.

Cluster 3 contained independent isolates from four continents and only strains from the same countries shared high sequence identities (e.g., 92% among Irish and 98% among Hungarian strains).

Cluster 4 was the most genetically diverse group of chicken reoviruses and contained the largest number of isolates from our global collection. Two major subclusters were distinguished and both included multiple strains from all four continents where strains originated from. Some lineages included closely related European strains from different countries and study years, mainly from early 2010's. Close genetic relatedness was seen, for example, among some German strains (96 to 99%, most probably representing strains of close geographic origin) and Malaysian strains (95%).

**Figure 6:**
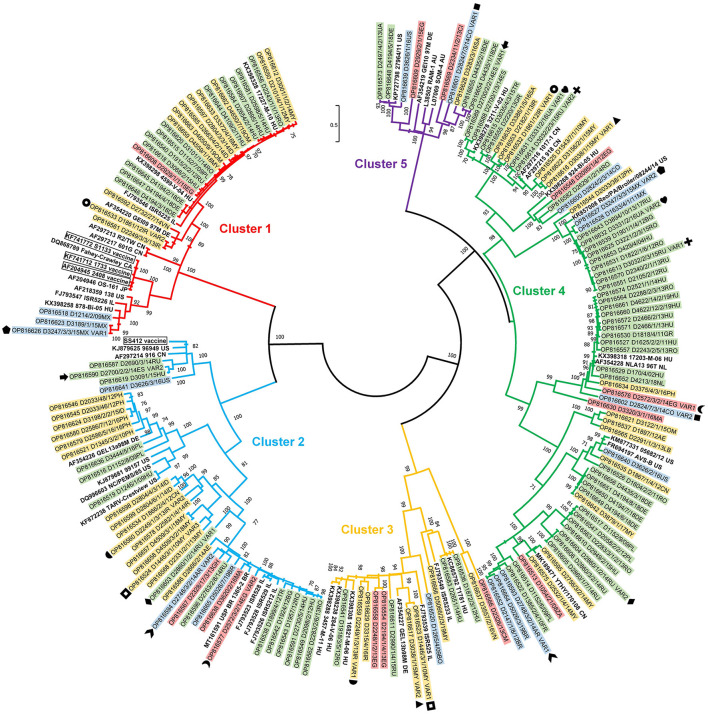
Phylogenetic tree of detected ARV strains based on the partial amino acid sequence coded by the σC gene.

Cluster 5 had the lowest number of isolates and only some German and Ukrainian isolates shared high sequence identities (98%) (Figure 6).

Strains belonging to each of five clusters were isolated from both ARV specific clinical conditions: tenosynovitis cases: 9 (15%), 4 (7%), 1 (2%), 12 (20%), 4 (7%) isolates for cluster 1, cluster 2, cluster 3, cluster 4 and cluster 5, respectively; runting-stunting syndrome: 2 (6%), 7 (19%), 1 (3%), 10 (31%), 3 (9%) isolates of cluster 1, 2, 3, 4, and 5, respectively and 9 isolates (31%) with unidentified cluster (not sequenced) based on our dataset. In two cases, cluster 2 and cluster 4 strains were identified from 3- and 5-weeks old broilers, reportedly showing the clinical signs of both tenosynovitis and runting-stunting syndrome simultaneously.

**Figure 7:**
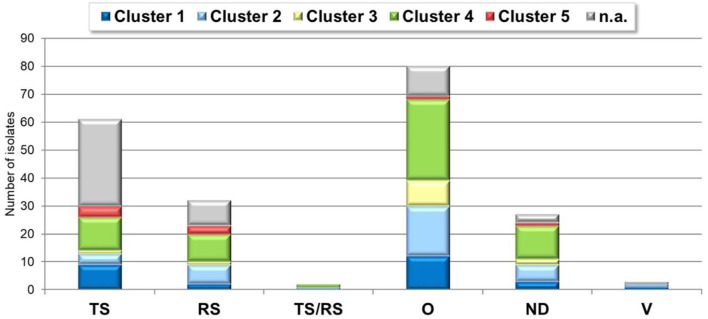
ARV cluster-clinical condition distribution. TS, tenosynovitis/arthritis; RS, runting-stunting syndrome; O, other; ND, not defined; V, vaccine.

About half of detections, 107 isolates (52% of the total sample number) could not be linked to specific ARV syndromes. Representatives of each cluster could be found in the phylogenetic analysis of these strains. Remarkably, in case of 27 isolates (13% of the submitted cases) no disease history of the flocks was disclosed (Figure 7).

Vaccine related or vaccine derived sequences were not identified among the isolates obtained from field cases. Most of the vaccine strains fell in cluster 1, the only exception was ss412 that belongs to Cluster 2. The closest genetic similarity between a study strain and a vaccine strain was 77%. Cluster 1 contained multiple closely related strains (range 94 to 98%) from European, Central and South Asian, and North African countries. A separate lineage within cluster 1 contained some Central and North American strains sharing 86 to 100% pairwise identities.

## 4. Discussion

Avian reovirus infections in chickens poses a major challenge to poultry industry worldwide. The knowledge about the circulating strains is of great importance to improve the related control measures. Our strain collection, being built over a decade already and covering 34 countries at present, provides an overview on this aspect, however, the prevalence of the ARV related diseases in the different geographical areas could not be estimated due to the low number of samples.

Viral arthritis and runting-stunting syndrome are the clinical manifestations which are directly connected the ARV, but poor feed conversion ratio, increased condemnation in slaughterhouses due to secondary bacterial infections are indirect, but very important effects of this virus causing marked economical losses (21, 22). About half of the ARV isolates included in our study were obtained from tenosynovitis/arthritis or runting-stunting syndrome, while the other half originated from different types of clinical conditions such as respiratory or egg production problems, gout, kidney lesions or increased bacterial infections.

In one third of the cases, ARV was detected together with one or more other infectious agents.

While arthritis/tenosynovitis occurred both in the early ages and in older breeders too, runting-stunting syndrome was present from 1 to 8 weeks of age based on our database. The lack of appropriate metadata (clinical/treatment history, necropsy findings) prevented the proper interpretation of co-infections in the case of a few submissions.

The phylogenetic analysis of a representative ARV strain collection indicated the higher divergence rate of the S1 genome segment (encoding the σC protein) than the other S-class genes (23).

Sequence analysis of a portion of the S1 gene segment has been commonly used for the characterization and classification of ARV isolates, but other genome segments were also targeted with similar objectives (24, 25). Historically, partial S1 gene characterization methods have classified ARV strains into five genotypic clusters, but more recent works attempted to introduce additional genetic clusters (8, 10, 12, 26–28). However, these approaches were based on different sets of sequences and different methodology, which may lead to controversy in the classification of some branches within the obtained tree topology. Due to these shortcomings, we applied the original classification scheme by Kant et al. (7). Nevertheless, our results also demonstrated a diversity beyond the established system, which further justifies the need to establish a generally accepted, uniform, robust, molecular ARV ^(1, 2)^ typing scheme, most probably including several genome segments.

In alignment with the above, we sequenced the partial σC gene of 136 ARV isolates and classified into the five major clusters (7). All the five clusters were represented in Asia, Europe, America and Africa based on our dataset, despite the fact that the number of ARV strains arrived from America or Africa were significantly lower compared to the numbers arrived from the other continents. There was no direct connection between the σC gene phylogenetic classification of the isolates and the clinical symptoms observed in the field, a finding that is echoes previous observations (7, 23, 26, 29). Although σC is responsible for cell attachment and serve as a primary mediator in tissue and organ tropism, viral determinants other than σC may also play a role in viral pathogenicity. Large-scale whole genome sequencing of ARV strains could be a useful approach to uncover missing connection between strain diversity and pathogenic features.

Novel variant stains are reported to cause vaccine breakthrough in vaccinated breeder or broiler flocks (5, 10, 13, 27). The inefficient protection induced by commercially available vaccines in these flocks is explained by the difference of σC gene between the vaccine and field challenge strains and the consequent inefficient serological cross-reaction in immunized birds against the new viruses (5). In most submissions of the presented collection only a single ARV strain was isolated, but there were 13 clinical cases where two or three ARV strains were identified. These mixed infections may result in the emergence of new variant strains [primarily due to reassortment (9, 15)] which are able to cause outbreaks or vaccine breakthrough even in the immunized flocks.

Next, a selection of the presented ARV strains will further be characterized for their antigenic and pathogenic properties. The obtained information will support more efficient vaccine strain selection in control efforts against ARV infections.

## Data availability statement

The datasets presented in this study can be found in online repositories. The names of the repository/repositories and accession number(s) can be found below: NCBI GenBank [https://www.ncbi.nlm.nih.gov/genbank/], OP816513-OP816738.

## Author contributions

EK conceptualized and drafted the manuscript, analyzed the data, and prepared the figures. RV-K and SF performed sequencing, analyzed the sequence data, and drafted the manuscript. TM and ZH participated in the laboratory investigations and contributed to the materials and methods chapters. TT-K participated in the data analysis and contributed to the manuscript draft. IK conceptualized, initiated, and edited the manuscript. KB conceptualized, edited the manuscript, and participated in the sequence analysis. VP managed sample collection, test results, data interpretation, and edited the manuscript.
